# Study of Exhaled Nitric Oxide in Subjects with Suspected Obstructive Sleep Apnea: A Pilot Study in Vietnam

**DOI:** 10.1155/2016/3050918

**Published:** 2016-01-13

**Authors:** Sy Duong-Quy, Thong Hua-Huy, Huyen-Tran Tran-Mai-Thi, Nhat-Nam Le-Dong, Timothy J. Craig, Anh-Tuan Dinh-Xuan

**Affiliations:** ^1^Bio-Medical Research Center, Lam Dong Medical College, Dalat, Lam Dong 063, Vietnam; ^2^Department of Respiratory Physiology, Cochin Hospital, Paris Descartes University, Sorbonne Paris Cité, 75014 Paris, France; ^3^Department of Technology and Biology, Dalat, Lam Dong 063, Vietnam; ^4^Department of Pulmonology, St. Elisabeth Hospital, 5000-5999 Namur, Belgium; ^5^Pennsylvania State University, 500 University Drive, Hershey, PA 17033, USA

## Abstract

*Background and Objective*. The concentration of exhaled nitric oxide (eNO), reflecting the activity of inducible NO synthase in airway epithelium, has been found to increase in patients with obstructive sleep apnea (OSA). This study aimed to measure eNO concentration in patients with suspected OSA and to correlate different eNO parameters with clinical and sleep apnea characteristics.* Methods*. In this cross-sectional study, all patients underwent in-lab overnight polysomnography (PSG) and eNO measurement using a method of multiple flow rates before and after PSG (pre- and post-PSG).* Results*. According to the result of PSG, 82 persons were divided into two groups: control subjects (*n* = 30; 54 ± 14 years) and patients with OSA defined as apnea-hypopnea index (AHI) ≥ 5/hour (*n* = 52; 53 ± 12 years). Body mass index (BMI) and neck and abdomen circumferences of OSA patients were significantly higher than those from control subjects. In OSA group, post-PSG alveolar NO concentration (CANO) (5.3 ± 1.9 ppb) was significantly higher than pre-PSG CANO (4.0 ± 1.7 ppb; *P* < 0.001). Significant correlations have been found between CANO and AHI (*P* < 0.001) and between CANO and nadir SpO_2_ (*P* < 0.05). The daytime CANO value of more than 4.1 ppb can be used to screen symptomatic subjects for the presence of OSA with a high specificity of 93.3%.* Conclusion*. Our findings indicate CANO as a surrogate marker for OSA in persons with suggestive symptoms.

## 1. Introduction

Obstructive sleep apnea (OSA) is a chronic respiratory disorder with a high and increasing prevalence [1]. OSA is characterized by intermittent upper airway collapse and reopening during sleep, leading to intermittent hypoxemia (IH) and cardiovascular comorbidities. IH during sleep increases the production of reactive oxygen species and proinflammatory mediators. Oxidative stress and inflammation generate endothelial dysfunction, cardiovascular diseases such as arterial hypertension, coronary ischemia, and lung inflammation. Therefore, noninvasive assessment of lung inflammation may be useful to predict patients with OSA and its severity [2].

In humans, nitric oxide (NO) plays an important role in modulating vascular tone and airway inflammation. In the airway, NO can be easily measured in exhaled air in order to diagnose and evaluate the severity of chronic bronchial and alveolar inflammation [3]. The exhaled fraction of NO (FENO), reflecting total NO production in the airway, has been found to increase in patients with OSA [4–10]. Bucca et al. showed that complete tooth loss favoured upper airway obstruction and inflammation, as evidenced by increased eNO concentration with offline method [5]. In overweight children, habitual snoring and OSA, but not obesity alone, are associated with increased airway inflammation [6]. Later findings showed that FENO was correlated with OSA severity and decreased after positive pressure therapy [8–10].

However, FENO does not indicate the amount of NO being released from the periphery of the lung, that is, in bronchioles, alveoli, and interstitial spaces. Measurement of exhaled NO (eNO) using the two-compartment model (alveolar and bronchial) with multiple expiratory flow rates allows the determination of maximum bronchial NO flux (J'awNO) and alveolar NO concentration (CANO) by mathematical formula [10–12]. Therefore, CANO may be used to evaluate the inflammation of distal lung due to oxidative stress [12].

This study employed a mathematical formula [13] capable of discerning between bronchial and alveolar concentration to explore which one was better correlated with clinical symptoms and sleep apnea parameters. We measured the concentration of eNO in patients with suggestive symptoms of OSA and correlated different eNO parameters with clinical symptoms and sleep apnea characteristics.

## 2. Patients and Methods

### 2.1. Patients

Subjects living in Dalat city, Vietnam, who came to the Clinical Research Center of Lam Dong Medical College for screening of OSA, were included in this cross-sectional and case-control study. Subjects with acute and chronic cardiorespiratory diseases (acute myocardial infarction, severe coronary disease, chronic heart failure, COPD, and asthma) or diseases treated with local or systemic corticosteroids (allergic rhinitis and/or conjunctivitis) were excluded from the study.

All study subjects completed a screening questionnaire about symptoms of OSA, sleep habits and quality, and snoring. Epworth score was calculated for each patient (from 0 to 24). They underwent exhaled NO measurement and overnight polysomnography (PSG). Subjects were then randomized into two groups according to the results of PSG. Subjects with OSA were defined by apnea-hypopnea index (AHI) ≥ 5/hour and classified as having mild (AHI = 5–15), moderate (AHI = 16–30), or severe (AHI > 30) OSA. Subjects with AHI < 5/hour were included in control group.

### 2.2. Methods

#### 2.2.1. Polysomnography

In-laboratory overnight PSG was performed for each study subject using Alice 6 PSG (Philips, USA) as recommended [14]. The recording time was from 10 pm to 6 am of the day after. The minimum recording time for PSG was 6 hours with sleep time of at least 3 hours. Sleep G3 software was used to analyze PSG results. The recorded parameters were electroencephalography (EEG) with 4 channels: C4–A1, C3–A2, O2–A1, and O1–A2; chin electromyography (EMG); electrocardiography (ECG); nasal and buccal air flows; thorax-abdomen movements; sleeping posture; apnea-hypopnea index (times/minutes); type of apnea (central apnea, obstructive apnea, or mixed apnea); oxygen saturation (SpO_2_) and minimum SpO_2_ (nadir SpO_2_); arousal index (times/hour); snoring (>56 dB); and sleep efficiency (%).

#### 2.2.2. Exhaled NO

Exhaled nitric oxide was measured at multiple flow rates (50 mL/s, 100 mL/s, 150 mL/s, and 350 mL/s) before and after PSG (before PSG: 8 pm; after PSG: 6 am) using an electrochemical based analyzer (FeNO+, Medisoft-MGCD, USA). Technical measurement of exhaled NO was conducted according to manufacturer's instructions, as recommended by the ATS/ERS guideline [15]. The maximal bronchial production rate of NO (J'awNO) and alveolar concentration of NO (CANO) were automatically determined using the two-compartment model by Tsoukias and George [13]: VNO = J'awNO + CANO × VE via Expair's software.

### 2.3. Statistical Analysis

Data were analyzed using IBM-SPSS 22.0 software (Chicago, Illinois, USA). Values were expressed as mean ± standard deviation and 95% CI for quantitative variables and percentage for qualitative variables. Comparisons between the OSA patients and control subjects were done using Student's *t*-test. Pearson's chi-squared test (or Fisher exact test) was performed for verifying the relationship between qualitative variables. Linear correlation was analyzed by Spearman's nonparametric method. The best cut-off value of CANO for OSA screening was determined by ROC curve analysis. The statistical significance was stated with *P* < 0.05.

## 3. Results

### 3.1. Characteristics of Study Subjects

#### 3.1.1. Anthropometric and Clinical Characteristics

From March to September 2014, 82 subjects were included in the present study. They underwent polysomnography (PSG) and were divided into two groups: control subjects (AHI < 5; *n* = 30; 54 ± 14 years; male/female: 1/1) and subjects with OSA (AHI ≥ 5; *n* = 52; 53 ± 12 years; male/female: 1.2/1). All anthropometric and clinical characteristics of the study subjects are presented in Table 1. There were no significant differences between the two groups for mean age, percentage of active smokers, and Epworth scores. BMI and neck and abdomen circumferences of subjects with OSA were significantly higher than those of control subjects. The results of pulmonary function test showed no significant difference (FEV_1_, FVC, FEV_1_/FVC, and TLC) between the two groups (Table 1).

#### 3.1.2. PSG Outcomes

The increases of AHI and arousal index and the decreases of mean SpO_2_ and nadir SpO_2_ in subjects with OSA were significantly more important than those in the control subjects (Table 1).

### 3.2. Exhaled Nitric Oxide Analysis

The results of exhaled NO measured before and after polysomnography (pre- and post-PSG) and PSG parameters are presented in Table 1. In the two groups, the levels of pre-PSG exhaled NO (FENO, J'awNO, and CANO) were within normal limits (9.4 ± 6.6 ppb, 26.6 ± 19.2 nL/min, and 2.2 ± 0.7 ppb, resp., for control subjects; 16.7 ± 11.4 ppb, 38.3 ± 26.1 nL/min, and 4.0 ± 1.7 ppb, resp., for patients with OSA). All the parameters of exhaled NO in subjects with OSA were significantly higher than those of the control subjects (Table 1).

**Figure 1:**
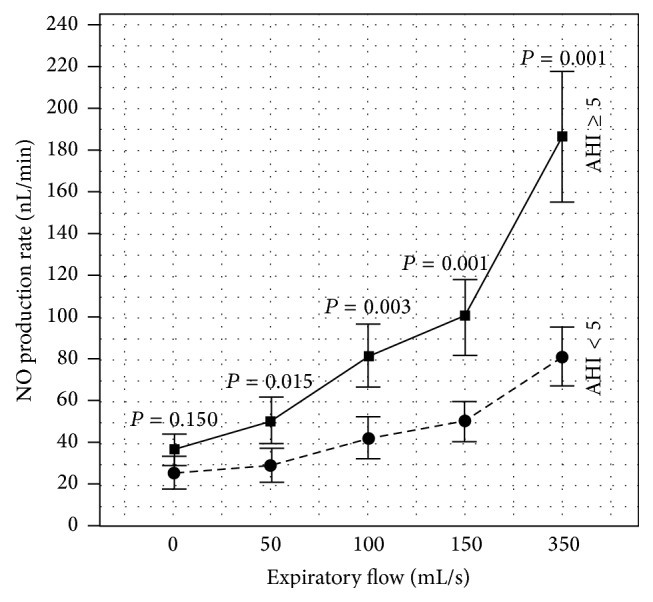
Post-PSG multiflow exhaled NO profile in two AHI groups. Note: the graph represents the mean (95% CI) of post-PSG NO production rate (VNO = FENO × Ex. flow), measured at 4 different levels of expiratory flow (50, 100, 150, and 350 mL/s). The J'awNO and CANO can be determined using the simple linear approach by Tsoukias and George [13]: VNO = J'awNO + CANO × Ex. flow. *P* value: significance of difference between healthy subjects (AHI < 5, circle point and dotted line) and patients with OSA (AHI ≥ 5, square point and continuous line).

Figure 1 presents the post-PSG NO profile according to the multiflow approach by Tsoukias and George [13]. As shown in this graph, the OSA patients have a significantly higher post-PSG CANO value (indicated by the slope of the regression curve) than the control group.

### 3.3. Changes in Exhaled NO before and after PSG

In the control group, there were a nonsignificant increase of J'awNO (31.7 ± 20.6 versus 26.6 ± 19.2; *P* = 0.197) and significant nonpathological increases of FENO at 50 mL/s and CANO after PSG versus before PSG (12.0 ± 5.9 ppb and 3.2 ± 1.1 ppb versus 9.4 ± 6.6 ppb and 2.2 ± 0.7 ppb; *P* < 0.01 and *P* < 0.001; resp.). In patients with OSA, there was a significant, nonpathological increase of FENO at 50 mL/s (22.1 ± 16.8 ppb versus 16.7 ± 11.4; *P* < 0.001) and a significant and pathological increase of CANO (5.3 ± 1.9 versus 4.0 ± 1.7 ppb; *P* < 0.001) after PSG versus those before PSG while there was no significant difference of J'awNO (39.1 ± 23.6 ppb after PSG versus 38.3 ± 26.1 ppb before PSG; *P* = 0.724) (Table 1).

### 3.4. Correlation between AHI, Clinical Sleep Apnea Markers, and Exhaled NO

#### 3.4.1. Correlations between AHI and Snoring, Epworth Score, and Exhaled NO

The correlations between AHI, snoring, Epworth score, and exhaled NO are presented in Table 2. There were no significant correlations in the control group (*P* > 0.05). In patients with OSA, there was a significant but slight correlation between AHI and FENO at 50 mL/s (*R* = 0.373, *P* = 0.007) and between AHI and J'awNO (*R* = 0.302, *P* = 0.03). Significant correlations have also been found between AHI and nadir SpO_2_ (*R* = −0.578, *P* < 0.001) and between AHI and CANO (*R* = 0.595, *P* < 0.0001; Table 2).

#### 3.4.2. Correlations between Post-PSG CANO and Snoring, Epworth Score, and PSG Parameters

**Figure 2:**
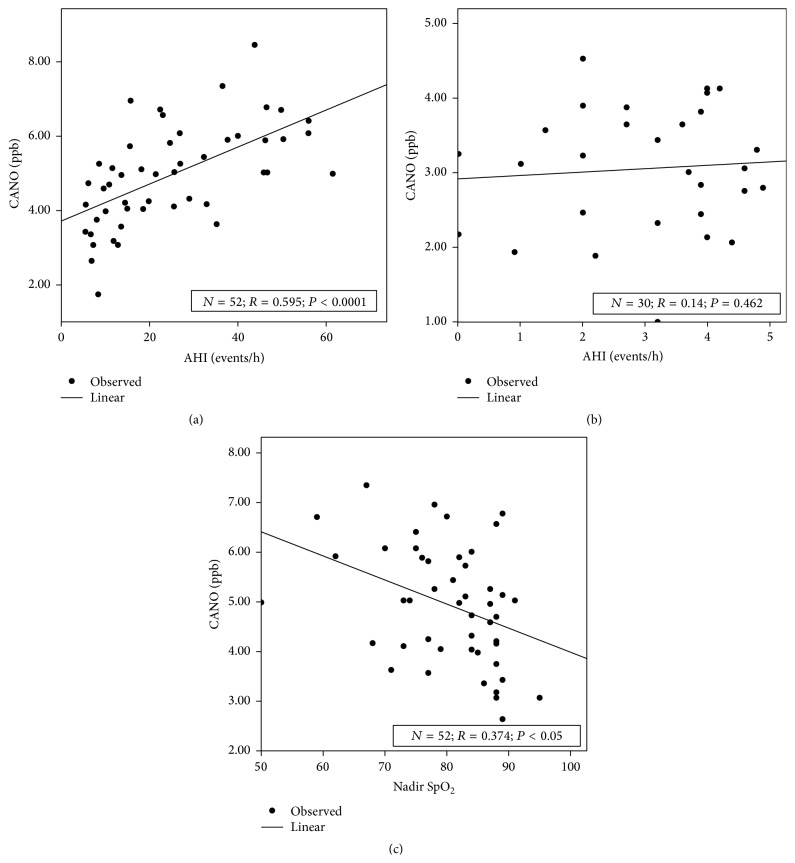
(a) Correlation between post-PSG CANO and AHI in subjects with OSA. There was a significant correlation between CANO and AHI (*R* = 0.595, *P* < 0.0001). CANO: alveolar concentration of nitric oxide; AHI: apnea-hypopnea index. (b) Correlation between post-PSG CANO and AHI in control subjects. There was no significant correlation between CANO and AHI (*R* = 0.073, *P* = 0.706). CANO: alveolar concentration of nitric oxide; AHI: apnea-hypopnea index. (c) Correlation between post-PSG CANO and nadir SpO_2_ in subjects with OSA. There was a significant correlation between CANO and nadir SpO_2_ (*R* = −0.374, *P* = 0.034). CANO: alveolar concentration of nitric oxide.

The correlations between post-PSG CANO and snoring, Epworth score, and PSG parameters are presented in Table 3. There were no significant correlations in the control group (*P* > 0.05; Figure 2(b)). In subjects with OSA, there was a moderate and significant correlation between CANO and AHI (*R* = 0.595, *P* < 0.0001; Figure 2(a)) and a slight correlation between CANO and nadir SpO_2_ (*R* = −0.374, *P* = 0.034; Figure 2(c)).

### 3.5. Cut-Off Point of CANO in Screening for Subjects with OSA

**Figure 3:**
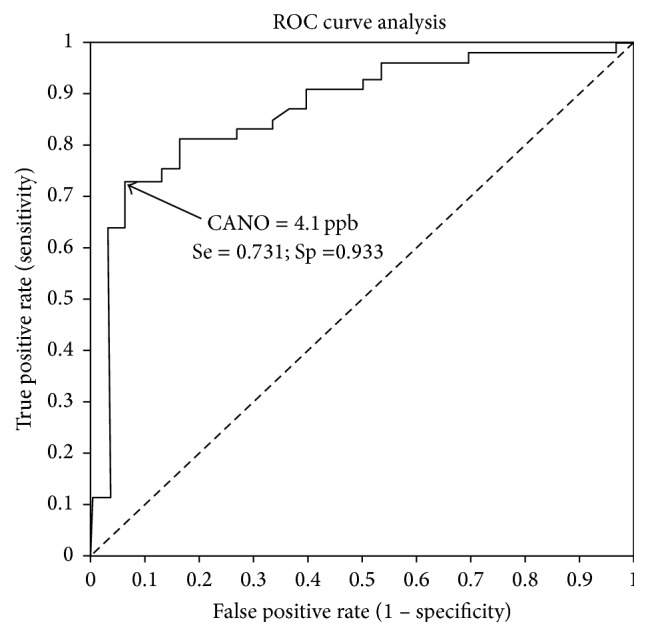
Receiver operating characteristic (ROC) curve of post-PSG CANO for predicting the subjects with OSA (AHI ≥ 5/h). CANO: alveolar nitric oxide concentration; PSG: polysomnography; post-PSG CANO: CANO measure on waking up after recording of PSG; Se: sensitivity; Sp: specificity. AUC: 0.868; best cut-off point: 4.1 ppb (Youden's *J* Index = 0.664) (see Table 4).

We performed the receiver operating characteristic (ROC) curve of post-PSG CANO for predicting the subjects with OSA (AHI ≥ 5 events/hour) and defined the best cut-off point at 4.1 ppb with highest Youden's *J* Index (0.664) (Figure 3). Sensibility, specificity, positive predictive value (PPV), and negative predictive value (NPV) were presented in Table 4 and Figure 3.

## 4. Discussion

The results of the present study showed that (1) the levels of exhaled NO (FENO, J'awNO, and CANO) during daytime were significantly higher in subjects with OSA than those without OSA; (2) FENO (at all expiratory flow rates) and CANO but not J'awNO were significantly increased on waking up (post-PSG) in both groups; and (3) significant increase of CANO in OSA subjects (as compared with control subjects) was correlated with the severity of OSA (AHI and nadir SpO_2_).

In the present study, all study subjects were divided into two groups depending on apnea-hypopnea index (AHI) measuring by polysomnography (PSG): subjects without OSA (AHI < 5) and subjects with OSA (AHI ≥ 5). Similar to previous studies [4–12], subjects with OSA had some risk factors for OSA such as high weight and BMI and increased neck and abdomen circumferences. However, in the present study, snoring and Epworth score were not significantly different between subjects with or free of OSA (Table 1). In addition, there were no significant correlations between AHI and snoring and Epworth score in subjects with OSA. Our result suggests that snoring and Epworth score are less sensitive for diagnosis of OSA and for prediction of the severity of OSA. Hence, finding out a new predictive marker of OSA severity seems to be necessary.

Recent studies showed that exhaled NO (eNO), especially the measure of alveolar concentration of NO (CANO) on waking up, may be useful to diagnose and predict the severity of OSA [7, 11, 12]. The results of our study showed that the levels of eNO (FENO, J'awNO, and CANO), measured during daytime in standard conditions in subjects with OSA, were significantly higher than in subjects without OSA (Table 1) but these values were still within the normal limits for healthy subjects. Therefore, we cannot use daytime eNO to diagnose subjects with OSA. Interestingly, although FENO and CANO, but not J'awNO, were significantly increased on waking up (post-PSG) in comparison with FENO and CANO measured in daytime (pre-PSG) in subjects free of OSA (Figure 2), there was only a significant and pathological increase of CANO after PSG in subjects with OSA (*P* < 0.001; CANO > 5 ppb; Figure 1). Thus, CANO might meet the criteria for a surrogate for OSA in this symptomatic population.

The concentration of eNO is changed during daytime but it must be within normal limits for healthy subjects (circadian rhythm) [16–18]. Previous studies demonstrated that eNO measured from the upper airway was significantly increased in subjects with OSA and that was linked to overexpression of inducible NOS (iNOS) [4, 5, 7, 8], which was partially reversible after CPAP treatment [8–10]. In our study, the level of FENO on waking up (post-PSG) was significantly increased in subjects with and without OSA (Figure 1) but this level was still within normal limits of healthy adults (<25 ppb). In addition, there was no significant correlation between FENO and AHI (Table 2). Therefore, FENO on waking up can not be used to predict OSA in suspected Vietnamese subjects.

The result of the present study shows that the level of CANO on waking up in subjects with OSA was significantly higher than that when they are awake and also higher than the normal limits (CANO > 5 ppb; Figure 1). Interestingly, there were significant correlations between post-PSG CANO, AHI, and nadir SpO_2_ (Tables 2 and 3 and Figures 2(a) and 2(b)), suggesting that CANO measurement on waking up may be useful to detect subjects at high risk of OSA. The result of our study showed that the specificity of CANO to diagnose OSA was best at 93.3% when the level of CANO exceeded 4.1 ppb. This result was similar to our previous study in European subjects with OSA, demonstrating the threshold of CANO at 4.5 ppb as the most relevant cut-off to specifically detect patients at high risk of OSA and nocturnal oxygen desaturation (NOD) among those with suggestive symptoms of OSA. Patients with NOD had more severe disease and might have suffered from more intense pulmonary inflammation, accounting for a higher cut-off value of CANO as compared to that found in this study. Another difference was the use of an electrochemical NO analyzer which is less expensive and more easily accessible for developing countries such as Vietnam [19].

Increased CANO in subjects with OSA has been found to be associated with oxygen desaturation during sleep [12, 20, 21]. Increasing evidence suggests the potential role of IH during sleep has been involved in an increase of CANO in OSA patients [21, 22]. IH during sleep in patients with OSA is a crucial cause of oxidative stress that activates the activity of inducible nitric oxide synthase (iNOS or NOS-2) to overproduce NO in alveolar epithelial cells. Although this study was conducted on a small number of subjects from a local population, the results of our study confirm for the first time in Vietnam that CANO is a useful biomarker in screening subjects at high risk of OSA. However, more studies in a large number of Vietnamese populations should be done to confirm the role of eNO in subjects with OSA.

## 5. Conclusion

Measurement of exhaled NO on waking up is useful to predict risk of OSA in subjects with suggestive symptoms. Alveolar exhaled NO (CANO) may be used as a surrogate marker for OSA.

## Figures and Tables

**Table 1:** Clinical and functional characteristics of study subjects.

Parameters	Control group (*n* = 30)		OSA (*n* = 52)		*P* value
	Mean ± SD	95% CI	Mean ± SD	95% CI	
*Clinical characteristics*					
Age, years	54.23 ± 14.19	48.93–59.50	53.98 ± 12.81	50.72–57.13	0.936
Height, cm	158.77 ± 7.48	156.16–161.24	161.08 ± 6.90	159.32–163.07	0.171
Weight, kg	54.33 ± 7.94	51.73–56.99	62.31 ± 12.04	59.24–65.46	**0.001**
BMI, kg/m^2^	21.58 ± 2.98	20.58–22.60	23.85 ± 3.43	22.96–24.78	**0.002**
Neck._CIR_, cm	32.43 ± 2.71	31.54–33.27	35.23 ± 3.92	34.23–36.23	**<0.001**
Abd._CIR_, cm	78.60 ± 8.28	75.47–81.40	86.96 ± 10.16	84.13–89.72	**<0.001**
Epworth score	10.10 ± 4.54	8.00–11.50	10.83 ± 6.05	10.00–13.00	0.539
Gender, M : F	1 : 1		1.2 : 1		NS
Smoker, %	10.0		13.5		NS
Snoring, %	76.7		76.9		NS
*Lung function *					
FEV_1_, % pred.	89 ± 11	85.06–92.94	88 ± 12	84.74–91.26	0.96
FVC, % pred.	91 ± 10	87.42–94.58	89 ± 11	86.01–91.99	0.90
FEV_1_/FVC, %	78 ± 7	75.49–80.50	79 ± 8	76.83–81.17	0.93
TLC, % pred.	94 ± 8	91.13–96.86	94 ± 8	91.83–96.17	1.00
*PSG parameters*					
TST, min	531.94 ± 62.43	508.35–553.04	523.74 ± 59.85	506.39–539.29	0.563
AHI, *n*/h	3.03 ± 1.39	2.46–3.51	25.61 ± 15.94	21.80–29.74	**<0.001**
ARI, *n*/h	23.37 ± 15.39	17.67–28.69	37.10 ± 18.83	31.94–42.58	**0.001**
SpO_2_, %	92.43 ± 2.51	91.57–93.30	88.67 ± 4.20	87.52–89.73	**<0.001**
Nadir SpO_2_, %	89.93 ± 3.29	88.80–91.03	79.83 ± 9.31	77.27–82.08	**<0.001**
*Before PSG*					
FENO at 50 mL/s, ppb	9.4 ± 6.6	7.5–12.0	16.7 ± 11.4	14.2–20.0	**0.003**
FENO at 100 mL/s, ppb	6.9 ± 4.0	5.7–8.3	13.1 ± 8.0	11.1–15.3	**0.002**
FENO at 150 mL/s, ppb	5.6 ± 2.6	4.7–6.6	11.3 ± 6.7	9.6–13.3	**0.001**
FENO at 350 mL/s, ppb	3.9 ± 1.6	3.3–4.5	8.6 ± 4.7	7.4–9.9	**0.001**
J'awNO, nL/min	26.6 ± 19.2	20.6–33.5	38.3 ± 26.1	31.5–45.2	**0.028**
CANO, ppb	2.2 ± 0.7	1.9–2.4	4.0 ± 1.7	3.5–4.5	**0.001**
*After PSG*					
FENO at 50 mL/s, ppb	12.0 ± 5.9	10.0–14.3	22.1 ± 16.8	18.2–26.9	**0.015**
FENO at 100 mL/s, ppb	8.9 ± 3.6	7.6–10.3	17.3 ± 11.3	14.7–20.5	**0.003**
FENO at 150 mL/s, ppb	7.4 ± 2.9	6.4–8.6	15.3 ± 8.5	13.0–17.9	**0.001**
FENO at 350 mL/s, ppb	5.4 ± 2.2	4.6–6.2	11.2 ± 5.3	9.8–12.7	**0.001**
J'awNO, nL/min	31.7 ± 20.6	25.0–39.0	39.1 ± 23.6	33.1–45.4	0.150
CANO, ppb	3.2 ± 1.1	2.8–3.6	5.3 ± 1.9	4.8–5.8	**0.001**

Note: OSA: obstructive sleep apnea; BMI: body mass index; Neck._CIR_: neck circumference; Abd._CIR_: abdomen circumference; M : F: male : female; FEV_1_: forced expiratory volume in 1 second; FVC: forced vital capacity; TLC: total lung capacity; PSG: polysomnography; TST: total sleep time; AHI: apnea-hypopnea index; ARI: arousal index. FENO: fractional exhaled nitric oxide (measured at expiratory flows of 50, 100, 150, and 350 mL/s); CANO: alveolar nitric oxide concentration; J'awNO: maximal bronchial flux of nitric oxide (using the two-compartment model). 95% CI: bootstrap confidence interval is based on 1000 replications; NS: no significant difference.

**Table 2:** Correlations between AHI and snoring, Epworth score, and exhaled NO.

Parameters	AHI < 5 (*n* = 30)	AHI ≥ 5 (*n* = 52)
Snoring	*R* = −0.084, *P* = 0.654	*R* = −0.216, *P* = 0.125
Epworth score	*R* = −0.085, *P* = 0.654	*R* = −0.021, *P* = 0.883
Nadir SpO_2_, %	*R* = 0.222, *P* = 0.237	*R* = −0.578, **P** ** < 0.001**
FENO at 50 mL/s, ppb	*R* = 0.074, *P* = 0.699	*R* = 0.373, **P** ** = 0.007**
J'awNO, nL/min	*R* = 0.272, *P* = 0.146	*R* = 0.302, **P** ** = 0.030**
CANO, ppb	*R* = 0.140, *P* = 0.462	*R* = 0.595, **P** ** < 0.0001**

Note: AHI: apnea-hypopnea index; FENO: fractional exhaled nitric oxide (measured at expiratory flows of 50, 100, 150, and 350 mL/s); CANO: alveolar nitric oxide concentration; J'awNO: maximal bronchial flux of nitric oxide (using the two-compartment model).

**Table 3:** Correlations between CANO and snoring, Epworth score, and PSG parameters.

Parameters	Control subjects (*n* = 30) CANO = 3.2 ± 1.1	OSA subjects (*n* = 52) CANO = 5.3 ± 1.9
Snoring	*R* = −0.040, *P* = 0.833	*R* = −0.150, *P* = 0.287
Epworth score	*R* = −0.305, *P* = 0.101	*R* = −0.187, *P* = 0.184
AHI, *n*/h	*R* = 0.140, *P* = 0.462	*R* = 0.595, **P** ** < 0.0001**
SpO_2_, %	*R* = −0.166, *P* = 0.381	*R* = −0.153, *P* = 0.278
Nadir SpO_2_ (%)	*R* = −0.215, *P* = 0.254	*R* = −0.374, **P** ** = 0.034**
Arousal index	*R* = −0.102, *P* = 0.592	*R* = −0.207, *P* = 0.141

CANO: alveolar nitric oxide concentration; PSG: polysomnography; OSA: obstructive sleep apnea; AHI: apnea-hypopnea index.

**Table 4:** Statistical parameters for the best cut-off point of CANO (4.1 ppb).

	Estimated value	95% CI	
		Lower bound	Upper bound
Sensitivity	0.731	0.597	0.832
Specificity	0.933	0.787	0.981
PPV	0.950	0.835	0.986
NPV	0.667	0.516	0.790
LR+	10.962	2.844	42.243
LR−	0.288	0.182	0.456
Accuracy	0.805	0.706	0.876

PPV: positive predictive value; NPV: negative predictive value; LR: likelihood ratio; CI: confidence interval.
